# Mechanical and Drying Shrinkage Performance Study of Ultra-High-Performance Concrete Prepared from Titanium Slag under Different Curing Conditions

**DOI:** 10.3390/ma17174201

**Published:** 2024-08-25

**Authors:** Jinxin Wang, Jun Li, Yan Gao, Zhongyuan Lu, Li Hou

**Affiliations:** 1 State Key Laboratory of Environment-Friendly Energy Materials, School of Materials and Chemistry, Southwest University of Science and Technology, Mianyang 621010, China; swustwangjinxin@163.com (J.W.); luy@swust.edu.cn (Z.L.); 2 Department of Materials Engineering, Sichuan College of Architectural Technology, Deyang 618000, China; gaoyan1516@126.com; 3 School of Civil Engineering and Architecture, Southwest University of Science and Technology, Mianyang 621010, China; houli@swust.edu.cn

**Keywords:** low-heat cement, steam curing, hydration products, pore structure optimization, drying shrinkage reduction

## Abstract

This research investigates the effects of various curing regimes, the incorporation of titanium slag, and the utilization of quartz sand on the strength properties and shrinkage behavior of ultra-high-performance concrete (UHPC). By using low-heat silicate cement to prepare UHPC, this study conducted standard curing and steam curing, and comprehensively analyzed the macro and micro performance of UHPC under different curing conditions. The findings indicate that the application of steam curing markedly enhances the mechanical attributes of UHPC while efficiently decreasing its drying shrinkage. In the comparative tests, we found that the compressive strength of concrete that had undergone 2 days of steam curing was 9.15% higher than that of concrete cured for 28 days under standard conditions. In addition, under the same curing conditions, titanium slag sand had higher mechanical properties than quartz sand. Under standard curing conditions, the 28-day compressive strength of UHPC using titaniferous slag aggregate was 12.64% higher than that of UHPC using standard sand. Through the data analysis of XRD, TG, and MIP, we found that the content of Ca(OH)_2_ in the hydration products after steam curing was reduced compared to the standard curing conditions, and the pore structure had been optimized. The UHPC prepared with titanium slag sand has greater advantages in mechanical properties and drying shrinkage, and has a smaller pore structure than the UHPC prepared with quartz sand. Moreover, the use of titanium slag sand offers ecological and economic benefits, making it a more sustainable and cost-effective option for high-performance construction applications.

## 1. Introduction

UHPC is an innovative civil engineering material widely recognized for its exceptional strength and ductility [1,2,3,4]. These characteristics make UHPC particularly suitable for constructing blast-resistant structural elements [5,6], large-span bridges [7,8], and structures exposed to severe erosive environments [1,9]. Curing procedures are essential for the attainment of early strength in UHPC [10,11,12]. Thermal processing can expedite the hydration of cement clinker and the pozzolan reaction of the added minerals in UHPC, thereby enhancing the C-S-H gel content and demonstrating remarkable mechanical performance rapidly [12,13]. Research indicates that heat treatment can enhance the mechanical properties of UHPC, achieving a compressive strength of up to 250 MPa, a resistance in traction by inflection of 45 MPa and a tensile direct strength of approximately 20 MPa [14]. The large amount of cementitious materials in UHPC, often using quartz sand as aggregate [15], results in a large UHPC hydration heat, and the high cost and high energy consumption hinder its large-scale application. Although UHPC boasts exceptional mechanical attributes and resilience, its heavy reliance on Portland cement has sparked worries over environmental implications, particularly in terms of CO_2_ emissions. The amount of Portland cement used in UHPC is usually two to three times that of traditional concrete, resulting in more CO_2_ being produced during its production process than traditional concrete [1,16]. To solve this problem, existing research usually uses active admixtures such as fly ash, granulated blast furnace slag, and metakaolin to partially replace Portland cement [1,17,18,19]. A study has found that after incorporating 10% metakaolin, the 7-day compressive strength of UHPC increased by 20.1% [20]. In addition, the use of low-heat Portland cement is also considered an effective method to reduce CO_2_ emissions [12,21,22,23]. Low-heat Portland cement is mainly composed of dicalcium silicate (C_2_S) [24,25]; its heat of formation is about 1350 kJ/kg, which is about 25% lower than the main component of traditional cement, tricalcium silicate (C_3_S). When producing this cement, the temperature of firing clinker can be reduced from 1450 °C to between 1300 °C and 1350 °C. This change reduces energy consumption during production and also reduces carbon emissions, so it is considered a green low-carbon cement. It is noteworthy that low-heat Portland cement exhibits a relatively low temperature rise during the early stages of hydration [26,27]. Historical research has shown that the heat of hydration of low-heat Portland cement at 7 days is reduced by 18.12% compared to that of ordinary Portland cement [28]. Additionally, its chemical shrinkage rate is also lower than that of ordinary Portland cement [21,23,29]. Given these advantages, this paper considers the use of low-heat Portland cement for the preparation of UHPC. Although the early mechanical properties of low-heat Portland cement are lower, this defect can be accelerated by increasing the curing temperature to accelerate the hydration of cement, thereby improving its early mechanical properties.

Titanium slag sand is an industrial solid waste obtained by cooling the slag discharged from the smelting of iron ore in a blast furnace after the beneficiation of vanadium–titanium magnetite [30,31]. Titanium slag sand is mainly composed of titanium slag aggregate particles and some titanium slag micro-powder. The rough surface of the titanium slag aggregate particles can improve the interface structure between the aggregate particles and the slurry, and the micro-powder can improve the compactness of the cementitious material system [31,32].

In an innovative approach, this paper addresses the dual objectives of low CO_2_ emissions and superior UHPC performance by utilizing low-heat Portland cement and titanium slag sand. The study meticulously investigates the mechanical properties, drying shrinkage characteristics, and microstructure of UHPC formulated with titanium slag sand, employing both standard curing and steam curing methods to optimize the material’s properties.

## 2. Experimental

### 2.1. Material

**Figure 1:**
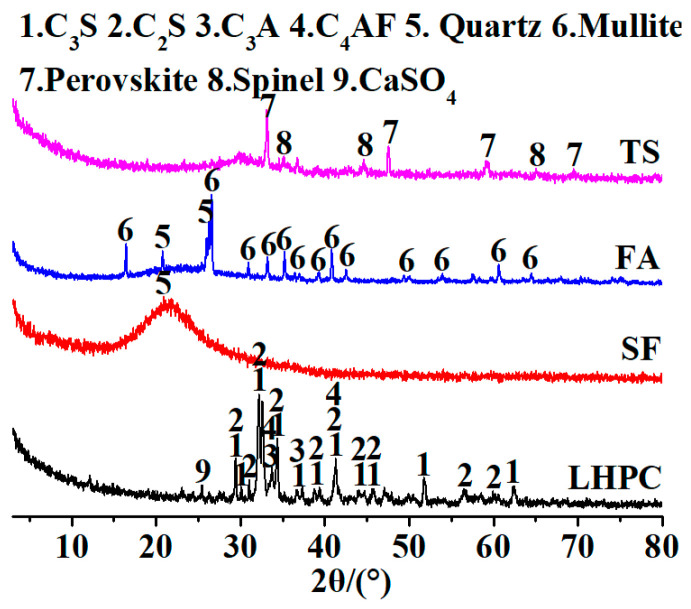
XRD patterns of LHPC, FA, TS, and SF.

The low-heat silicate cement (LHPC) used in this experiment was produced by Jiahua Special Cement Co., Ltd. (Leshan, China), and its physical properties are shown in Table 1. The fly ash (FA) was provided by Guoneng Sichuan Energy Co., Ltd. (Mianyang, China), Jiangyou power plant. The silica fume (SF) was produced by Sichuan iwind new material Co., Ltd. ( Chengdu, China),. The water reducing agent is provided by Tongzhou Chemical Technology Co., Ltd. (Mianyang, China), with a water reduction rate of 25% and a solid content of 40%. Table 2 and Figure 1 show the chemical composition and mineral composition of cement, silica fume, and fly ash determined by an X-ray fluorescence spectrometer (XRF) and X-ray diffractometer (XRD), respectively. In terms of aggregates, high titanium slag (TS) produced by Gangcheng Group Co., Ltd. (Panzhihua, China), was used, with a particle size of less than 4.75 mm. Standard sand (SS) was used as a comparison material. The standard sand was sourced from Xiamen ISO Standard Sand Co., Ltd. (Xiamen, China).

**Figure 2:**
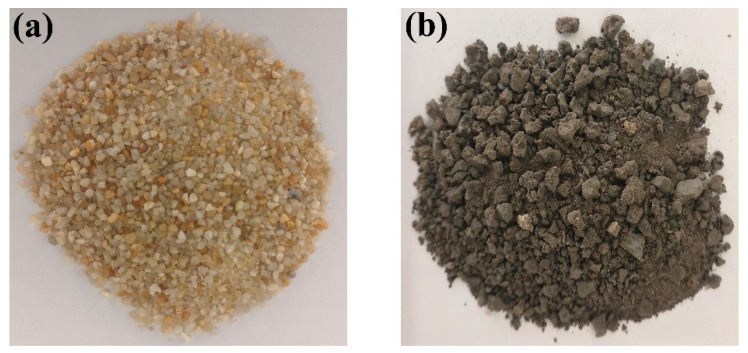
(**a**) Photos of the SS. (**b**) Photos of the TS.

Table 3 displays the comparative data of the cumulative sieve residue between standard sand and titanium slag sand. Figure 2 presents physical photographs of the two types of sand samples.

### 2.2. Sample Preparation and Maintenance

According to the mix proportions in Table 4, the method for preparing UHPC specimens is as follows: First, dry mix the LHPC, fly ash (FA), and silica fume (SF). Then, add high titanium slag sand or standard sand, and continue dry mixing for at least 2 min. Next, add water and a water reducer, mix evenly, and then discharge. Pour the concrete into a mold with dimensions of 40 × 40 × 160 mm^3^. It is important to note that a layer of plastic film should be placed on top of the prepared specimens to prevent moisture evaporation. This step is crucial and should be carried out at a room temperature of 20 °C. After 1 day of curing, the mold can be removed. This study used two different curing regimes: standard curing (SD), with a condition of 20 °C ± 2 °C and a humidity of 97% ± 2%, and steam curing (SM), using a temperature of 80 °C ± 2 °C, lasting for 48 h.

### 2.3. Test Methods

#### 2.3.1. Mechanical Flexural and Compressive Strength

According to the national standard GB/T 17671 [33], the mechanical properties of cement mortar are tested. The compressive strength of each sample is taken as the average of six cubic mortar specimens.

#### 2.3.2. XRD/XRF

The chemical composition of raw materials was tested by X-ray fluorescence (XRF, Axiox, PANalytical B.V., Almelo, The Netherlands). The mineral composition was characterized by an X-ray diffractometer (XRD, PANalytical X’PertPro, Almelo, The Netherlands) with a scanning angle range of 5° to 80° and a scanning rate of 10°/min. The hydration products of L-SD in standard curing at early (3 d) and late (28 d) stages, as well as L-SM in steam curing for 2 d, were tested.

#### 2.3.3. TG

The samples were subjected to weight-loss testing using a STA449F5 thermogravimetric analyzer (TG) manufactured by Germany Netzsch Instrument Manufacturing Co., Ltd. (Selb, Germany). The dried samples (20 ± 3 mg) were heated in flowing nitrogen gas (20 mL/min) within a temperature range of 30 °C to 1000 °C at a heating rate of 20 °C/min. The experimental samples consisted of L-SD, subjected to 3 days and 28 days of standard curing, and L-SM, which was steam cured for a period of 2 days.

#### 2.3.4. MIP

The solidified cement mortar was reduced to bits and submerged in absolute ethanol for 48 h to cease the hydration. Thereafter, the samples were dehydrated to a stable mass at 50 °C within a vacuum drying chamber, in anticipation of MIP analysis utilizing an Auto Pore IV 9500 device imported from the United States (Mike, Norcross, GA, USA). For the examination of the pore structure within the solidified cement mortar, particles measuring between 2 and 3 mm were chosen for the study. The samples used for the experiment were L-SD-SS and L-SD-TS, both undergoing standard curing for 3 and 28 days, in addition to L-SM-SS and L-SM-TS, which were subjected to steam curing for 2 days.

#### 2.3.5. Drying Shrinkage Teste

According to the experimental mix design, the molds were cast and leveled using a scraper. Immediately after molding, a layer of plastic film was applied to the surface of the mortar. The molds were then placed in a standard curing room for 24 h, after which they were demolded. Following demolding, the specimens were transferred to a drying shrinkage chamber where they were exposed to a temperature of 20 ± 2 °C and a relative humidity of 60 ± 5%. The samples were placed on non-absorbent shelves, elevated from the bottom, with a spacing of more than 30 mm between each specimen. The length of the specimens was measured at 1 day, 3 days, 7 days, 14 days, and 28 days, with three specimens tested for each mix and three repeat readings per specimen.

The formula for calculating the shrinkage rate (x) is as follows:

(1) $$x=\frac{L_{0}-L_{t}}{L_{b}}$$

where x represents the shrinkage rate at a specific time *t* (in days) following the initial length measurement (μm/m). *t* denotes the number of days that have elapsed since the initial length was recorded. *L_b_* is defined as the gauge length of the specimen, which is determined by measuring the distance between the inner edges of the two measurement probes, effectively capturing the specimen’s length while excluding the parts of the probes that extend beyond (mm). *L_0_* is the initial length measurement of the specimen (mm). *L_t_* is the length measurement of the specimen at time *t* (in days) (mm). The shrinkage rate is computed with a precision of 1.0 × 10^−6^.

## 3. Results and Discussion

### 3.1. Mechanical Properties

**Figure 3:**
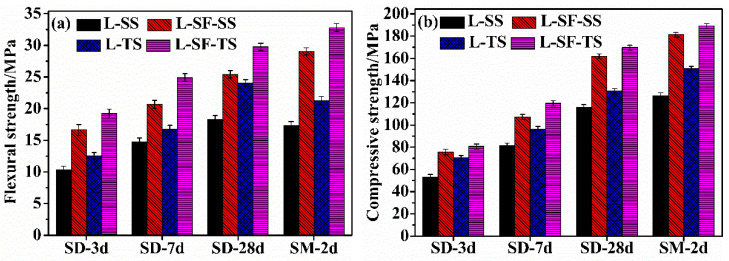
Mechanical properties: (**a**) flexural strength, (**b**) compressive strength.

Figure 3 illustrates the flexural and compressive strength of UHPC prepared with SS and TS aggregates under standard curing for 3 days, 7 days, 28 days, and steam curing for 2 days. From Figure 3, it can be observed that the mechanical performance of UHPC prepared with SS and TS aggregates gradually improves with increasing age under standard curing conditions. Furthermore, the addition of steel fibers enhances both the flexural and compressive strength of UHPC prepared with SS and TS aggregates under standard curing conditions, attributed to the beneficial contribution of steel fibers. Comparing the cases of L-SD-SS and L-SF-SD-SS, the flexural strength at 28 days increased from 18.3 MPa to 25.4 MPa, representing a 38.77% improvement. Similarly, comparing L-SD-TS and L-SF-SD-TS, the flexural strength at 28 days increased from 24.1 MPa to 29.8 MPa, showing a 23.46% improvement. Additionally, comparing L-SM-SS and L-SF-SM-SS, the flexural strength after 2 days of steam curing increased from 17.3 MPa to 29.1 MPa, demonstrating a 68.16% improvement. Similarly, comparing L-SM-TS and L-SF-SM-TS, the flexural strength after 2 days of steam curing increased from 21.3 MPa to 32.8 MPa, representing a 54.19% improvement. In the mechanical testing of UHPC, when the specimen reaches the initial cracking load, it reaches a critical point where cracks start to occur, eventually leading to failure. The addition of steel fibers in UHPC is analogous to reinforcing bars in conventional concrete, as they can bear some of the load and cause stress redistribution within the specimen. Even when cracks occur, steel fibers continue to bear the load, enabling the specimen to withstand greater loads and thereby improving the flexural strength of UHPC [34]. In UHPC, the incorporation of steel fibers primarily serves to induce a lateral confinement effect [35]. Consequently, when steel fibers are added to UHPC, they can significantly enhance its compressive strength, thereby demonstrating exceptional mechanical properties. By comparing the compressive strength of UHPC prepared with SS and TS aggregates, it is observed that, under both standard curing and steam curing conditions, UHPC prepared with TS aggregates exhibits higher compressive strength than that prepared with SS aggregates. This is primarily attributed to the denser interface between the TS aggregates and the cementitious matrix. The interface facilitates the formation of hydration products, and the TS aggregates exhibit physical interactions such as interlocking and embedding with the cementitious matrix [31]. Under steam curing conditions, UHPC demonstrates an improved mechanical performance compared to standard curing conditions. This is mainly due to the stimulation of reactivity in the presence of high-temperature curing, which promotes densification of the cementitious matrix by filling voids and reacting with the cement hydration product Ca(OH)_2_, resulting in the formation of a more compact hydrated calcium silicate (C-S-H) gel and an overall denser structure [11,36].

### 3.2. Drying Shrinkage

**Figure 4:**
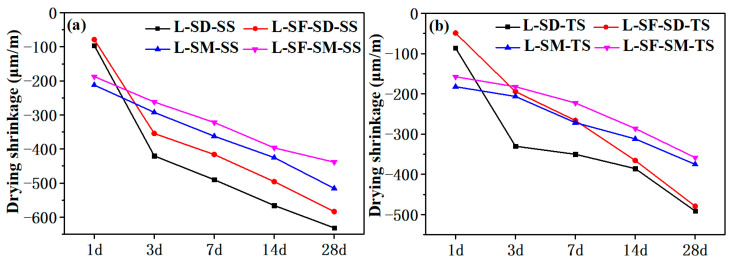
Drying shrinkage of UHPC. (**a**) Add to standard sand group; (**b**) Add titanium slag group.

Figure 4 illustrates the drying shrinkage rates of UHPC prepared with two types of aggregates over various periods of 1 day, 3 days, 7 days, 14 days, and 28 days in a drying chamber. The drying shrinkage of cement-based materials occurs due to internal moisture evaporation caused by lower external humidity compared to internal humidity. It is evident from Figure 4 that steam curing significantly reduces the drying shrinkage of concrete. Particularly noteworthy is the more pronounced inhibitory effect of steam curing on drying shrinkage after the addition of steel fibers [2]. Steam curing creates a high-temperature, high-humidity environment, promoting the activation of fly ash and silica fume to exhibit micro-aggregate and pozzolanic effects, resulting in the formation of dense C-S-H gel to fill voids [2,37]. This densifies the overall structure of UHPC, reduces porosity, and increases capillary negative pressure, thereby reducing the shrinkage rate. Moreover, due to the enhancement of the overall tensile strength of UHPC by steel fibers, tensile strength remains the dominant factor, resulting in a lower overall shrinkage rate [2]. In the high-humidity environment of steam curing, external steam presses into the concrete, with water molecule pressure exceeding capillary tensile stress, resulting in volume expansion that can partially counteract drying shrinkage [10]. Comparing the effects of the two aggregates on UHPC shrinkage, it is found that the shrinkage rate of UHPC-SS is greater than that of UHPC-TS. This is mainly because the porous structure and higher hardness of TS aggregates effectively constrain the deformation of cement paste and form hydration products at the interface with the paste, which is also a reason for the smaller shrinkage of TS aggregates [10,11,31].

### 3.3. XRD/TG

**Figure 5:**
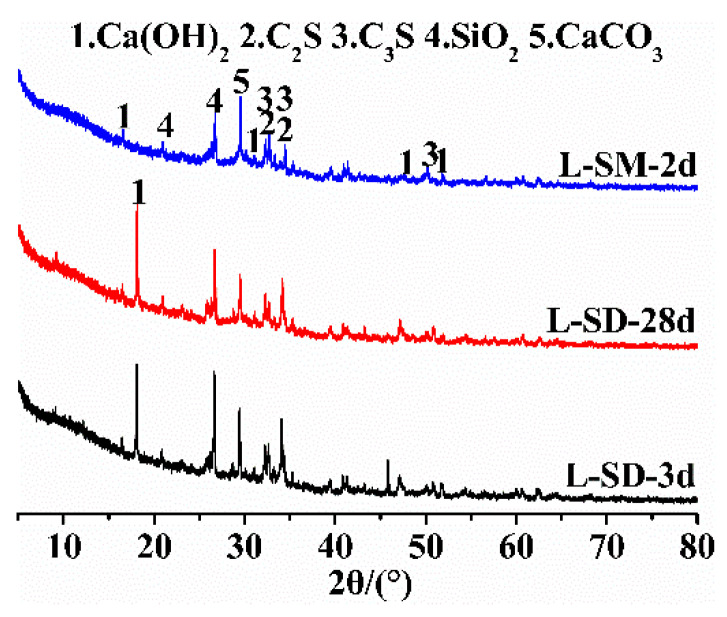
XRD pattern of hydration products.

The XRD patterns of the neat paste of UHPC under different curing regimes and curing times are presented in Figure 5. From the graph, it can be observed that the main hydration products are Ca(OH)_2_, SiO_2_, and CaCO_3_. Additionally, there are multiple peaks corresponding to C_2_S and C_3_S, indicating incomplete hydration due to the low water-to-cement ratio of the binder materials. Comparing the L-SD samples cured for 28 days under standard conditions with those cured for 3 days, a significant reduction in the peak intensity of Ca(OH)_2_ can be observed. As the curing age increases, the content of Ca(OH)_2_ should ideally increase; however, the presence of a higher amount of SF and FA in the system consumes the Ca(OH)_2_. The interaction between Ca(OH)_2_ and silica fume or fly ash triggers a secondary pozzolanic reaction that boosts the concrete’s density [11,36]. Therefore, a longer curing time leads to a greater consumption of Ca(OH)_2_. By comparing the two different curing methods, standard curing and steam curing, it is evident that the samples cured under standard conditions contain more Ca(OH)_2_ and unhydrated cement clinker (C_2_S, C_3_S), while the amount of Ca(OH)_2_ in UHPC paste significantly decreases after steam curing. This is attributed to the high temperature stimulating the pozzolanic activity of silica fume, promoting its secondary hydration reaction with Ca(OH)_2_, resulting in the consumption of Ca(OH)_2_ and the continuous production of new hydrated calcium silicate. Consequently, steam-cured UHPC exhibits higher strength compared to standard-cured UHPC. The XRD spectrum also shows peaks corresponding to calcium carbonate (CaCO_3_), which is a product of the reaction between Ca(OH)_2_ in the paste and CO_2_ in the environment, leading to the carbonation of Ca(OH)_2_ during sample preparation and testing.

**Figure 6:**
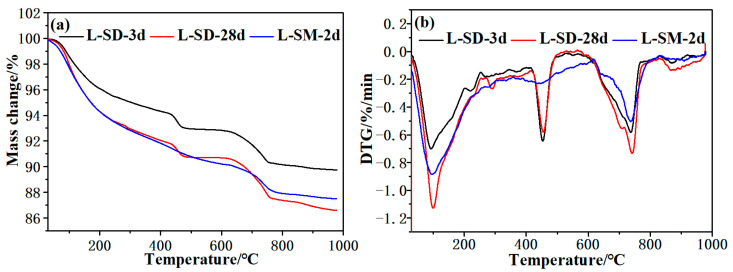
Thermal analysis curve of hydration products. (**a**) TG curves; (**b**) DTG curves.

Figure 6 presents the TG and DTG curves under different curing conditions. The TG curve shows a continuous decrease in sample mass with increasing temperature. The DTG curve reflects the changes that occur as the testing temperature increases, with different variations corresponding to different decomposition peaks. The weight loss between 30 °C and 105 °C is attributed to the evaporation of free water, while the mass loss between 105 °C and 400 °C is due to the dehydration of various gels. The DTG peak between 400 °C and 550 °C corresponds to the dehydroxylation of Ca(OH)_2_, and the DTG peak between 550 °C and 750 °C corresponds to the decarbonation of CaCO_3_ [38,39]. From Figure 6, it can be observed that a significant amount of hydrated calcium silicate is formed during the early hydration process, leading to an increase in the bound water content. The thermal dehydration causes noticeable mass loss. The dehydration of L-SD-28d is more pronounced than that of L-SD-3 d, indicating a higher degree of hydration with increasing age. During steam curing, the decrease in Ca(OH)_2_ content is mainly due to the accelerated rate of consumption of Ca(OH)_2_ by silica fume or fly ash in high-temperature environments. Similar to the XRD test results, the presence of CaCO_3_ indicates the carbonation of Ca(OH)_2_ in the samples during the preparation process and can reflect the Ca(OH)_2_ content in the samples.

### 3.4. Pore Structure

**Figure 7:**
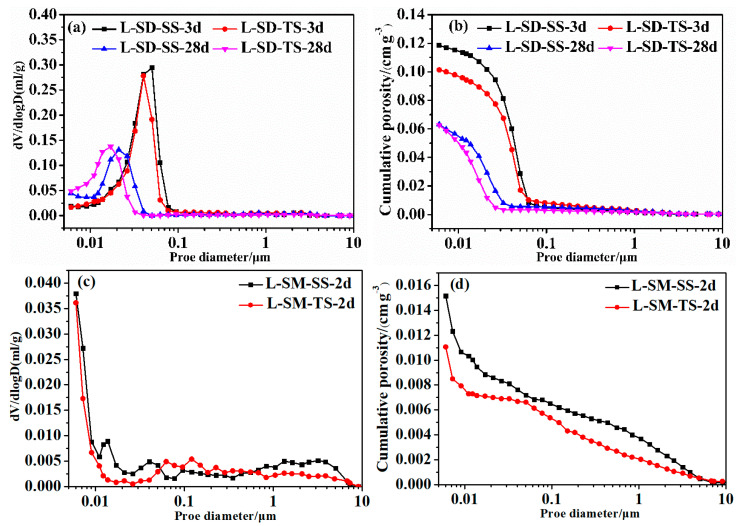
Pore size distribution curve of hydration products. (**a,b**) Pore structure of two kinds of sand under standard curing conditions; (**c,d**) Pore structure of two kinds of sand under steam curing condition.

As an important component of hardened paste, pore structure not only affects the compactness and physical properties of materials but also significantly influences their durability [21]. Additionally, the presence of numerous capillary pores in cement paste can lead to shrinkage stress during drying, resulting in cracking [21]. Taking into account the detrimental effects of pore dimensions on hardened paste, pores are categorized into four types: those less than 10 nm in size are termed as tiny capillary pores, and those falling within the 10 to 50 nm range are referred to as medium capillary pores, while those exceeding 50 nm are classified as large capillary pores [2,30,40]. Pores larger than 50 nm, often referred to as large capillary pores, have a certain impact on material permeability and strength, and the presence of large voids can easily form interconnected pores, reducing the material compactness and mechanical properties. To analyze the influence of two types of aggregates and two curing methods on the microstructure of UHPC pores, we conducted pore size distribution tests on mortar specimens cured under standard conditions for 3 d and 28 d and under steam curing for 2 d, as shown in Figure 7. The median pore sizes of UHPC prepared with SS and TS aggregates shifted towards smaller sizes with increasing hydration age. This is primarily because as cement hydration progresses, capillary voids are increasingly filled with hydration products, especially C-S-H and Ca(OH)_2_, leading to the refinement of pore structures [41,42,43]. It can be seen from Figure 7a,b, at 28 d, that UHPC prepared with TS aggregates exhibited smaller pore structures compared to that prepared with SS aggregates. As mentioned earlier, this is mainly because the porous structure of TS aggregates interacts physically with the cement paste, and TS aggregates contain a certain amount of TS micro-powder, which has a filling and nucleation effect on UHPC paste. According to Figure 7c,d, high-temperature environments have a significant impact on pore size distribution, with the median pore sizes of UHPC pastes made with both types of cement shifting towards smaller sizes. As mentioned earlier, steam curing can promote the further hydration of cementitious materials, increase structural compactness, and reduce large capillary pores. Comparing the pore size distributions of UHPC prepared with two types of aggregates under steam curing conditions, it was found that UHPC prepared with TS aggregates had smaller cumulative pores.

### 3.5. SEM

**Figure 8:**
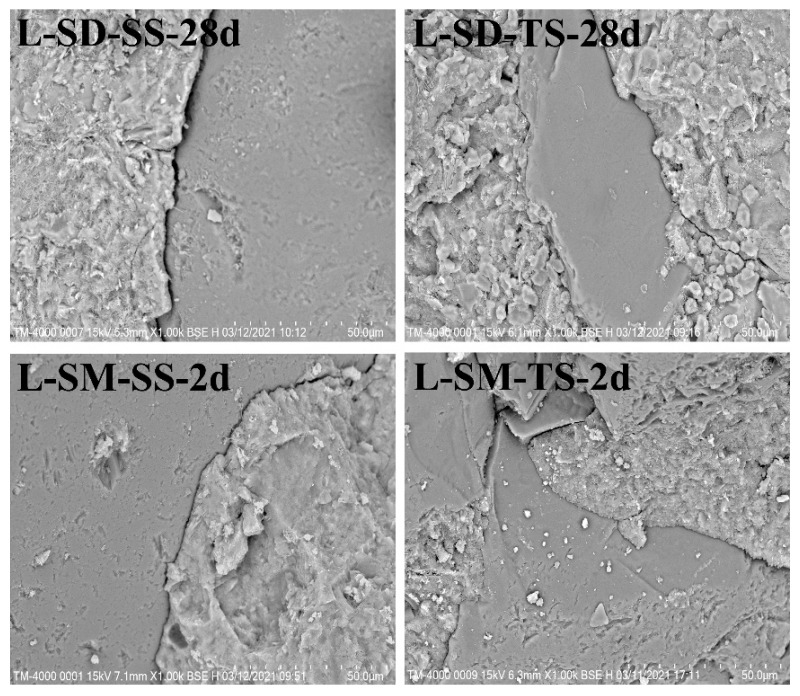
SEM images of different curing conditions and different sand.

The impact of two curing methods and two types of aggregates on the microstructure of UHPC prepared with low-heat Portland cement is demonstrated in Figure 8. Figure 8 presents the microstructure images of UHPC subjected to standard curing for 28 days and steam curing for 2 days. The photograph labeled L-SD-SS-28d clearly shows that, under standard curing, pronounced fissures are present between the aggregates made from standard sand and the cement matrix within the UHPC, suggesting a less dense connection between the aggregates and the cement paste. Conversely, in the image L-SD-TS-28d, it can be observed that the use of TS as aggregate leads to a reduction in cracks between the aggregates and the cement paste, primarily due to the presence of numerous pores on the surface of TS, resulting in a tighter bond.

A comparison between L-SD-SS-28d and L-SM-SS-2d reveals that under steam curing conditions, there is a tighter bond between the aggregates and the cement paste. This is mainly attributed to the rapid pozzolanic reaction between SF and cement under steam curing conditions, leading to a close bond between the aggregates and the cement paste surface. Similarly, comparing L-SD-TS-28d and L-SM-TS-2d shows a closer bond between the aggregates and the paste, which is the result of using TS aggregates under dual conditions of TS aggregates and steam curing. This outcome bears similarity to the pore structure in Figure 7 and further validates the relationship with mechanical properties from a lateral perspective.

## 4. Conclusions

This study investigates the effects of incorporating steel fibers and different aggregate types on the mechanical properties and microstructure of UHPC under various curing conditions. The findings reveal several key insights:

(1) Steel fibers enhance the flexural and compressive strengths of UHPC by impeding microcrack propagation, improving load transfer, and boosting load-bearing capacity. Titanium slag (TS) aggregates improve strength over silica sand (SS) aggregates by enhancing interfacial bonding with the cement paste. Steam curing surpasses standard curing by fostering advanced hydration and denser microstructure formation with increased C-S-H gel volume.
(2) Steam curing notably reduces UHPC drying shrinkage, especially with steel fibers, restraining crack development and deformation. UHPC with TS aggregates exhibits lower shrinkage due to compactness and reduced porosity from these aggregates.
(3) Steam curing enhances UHPC density by increasing hydration, consuming more Ca(OH)₂, and producing additional C-S-H gel. Elevated temperature and pressure accelerate hydration reactions, refining pore structure and densifying the matrix. TS aggregate UHPC boasts a finer pore distribution from improved packing and reduced porosity.
(4) TS aggregates in UHPC form a stronger bond with cement, reducing cracks and enhancing microstructure integrity, particularly under steam curing. TS aggregates’ rough texture aids in a robust transition zone, lessening microcrack formation and improving load transfer for a cohesive, durable microstructure.

## Figures and Tables

**Table 1:** Physical and mechanical properties of raw material.

Sample	Water Requirement of Normal Consistency (%)	Specific Grain Area (m^2^/kg)	Bulk Density (g/cm^3^)	Setting Time/min		Flexural Strength/MPa		Compressive Strength/MPa	
				Initial Setting Time	Final Setting Time	3d	28d	3d	28d
FA	-	458	0.89	-	-	-	-	-	-
SF	-	486	0.56	-	-	-	-	-	-
TS	-	-	1.18	-	-	-	-	-	-
LHPC	22.0	358	1.24	230	306	3.9	8.7	15.6	45.7

**Table 2:** Chemical compositions of raw materials (wt%).

Material	CaO	SiO_2_	Fe_2_O_3_	Al_2_O_3_	SO_3_	MgO	K_2_O	Na_2_O	LOI
LHPC	64.05	20.92	4.98	3.30	2.95	2.03	0.51	0.37	1.12
SF	0.46	97.29	0.05	0.25	0.71	0.59	0.34	0.02	2.53
FA	9.64	51.78	9.50	18.97	1.52	0.77	3.93	0.23	2.48
TS	26.14	23.52	4.11	13.25	1.25	8.14	0.12	0.38	0.00

**Table 3:** Particle gradation of standard sand and titanium slag sand.

Screen Size	Standard Sand	Titanium Slag Sand
	Cumulative Screening (%)	Cumulative Screening (%)
4.75	-	0.6
2.36	-	26.3
1.18	37.8	46.7
0.6	72.6	56.8
0.3	89.6	69.7
0.15	93.8	79.7
0.08	100.0	100.0

**Table 4:** UHPC mix ratio (kg/m^3^).

Specimen No.	LHPC	SF	FA	W/C	Water Reducing Agent	TS	SS	Steel Fiber (SF)
L-SD-TS	515.19	85.8	257.66	0.2	13	1683.52		
L-SF-SD-TS	515.19	85.8	257.66			1683.52		120
L-SM-TS	515.19	85.8	257.66			1683.52		
L-SF-SM-TS	515.19	85.8	257.66			1683.52		120
L-SD-SS	515.19	85.8	257.66				1683.52	
L-SF-SD-SS	515.19	85.8	257.66				1683.52	120
L-SM-SS	515.19	85.8	257.66				1683.52	
L-SF-SM-SS	515.19	85.8	257.66				1683.52	120

## Data Availability

The raw data supporting the conclusions of this article will be made available by the authors on request.
